# Exploring the Structure–Activity Relationships and Molecular Mechanisms of Black Soldier Fly-Derived Antimicrobial Peptides with AI Insights

**DOI:** 10.3390/insects17020207

**Published:** 2026-02-15

**Authors:** Muhammad Raheel Tariq, Hui Wang, Shaojuan Liu, Ilaria Armenia, Gianluca Tettamanti, Shakal Khan Korai, Haiwen Lin, Chaozhong Zheng, Yanwen Liang, Jianguang Qin, Youming Liu, Muhammad Qasim, Muhammad Asif Ismail, Fei Wang

**Affiliations:** 1 Agricultural Products Processing Research Institute, Chinese Academy of Tropical Agricultural Sciences, Zhanjiang 524001, China; raheeltariq916@gmail.com (M.R.T.); whqz1985@163.com (H.W.); liushaojuan2023@163.com (S.L.); linhw920@163.com (H.L.); realczzheng@163.com (C.Z.); liangyanwen0520@163.com (Y.L.); 2 College of Food Science and Technology, Huazhong Agricultural University, Wuhan 430070, China; lym@mail.hzau.edu.cn (Y.L.); asiffmian@gmail.com (M.A.I.); 3 Department of Biotechnology and Life Sciences, University of Insubria, 21100 Varese, Italy; ilaria.armenia@uninsubria.it (I.A.); gianluca.tettamanti@uninsubria.it (G.T.); 4 Interuniversity Center for Studies on Bioinspired Agro-Environmental Technology (BAT Center), University of Napoli Federico II, 80055 Portici, Italy; 5 Department of Grassland Science, College of Animal Science and Technology, Yangzhou University, Yangzhou 225009, China; khanshakal7@gmail.com; 6 College of Science & Engineering, Flinders University, Adelaide, SA 5001, Australia; jian.qin@flinders.edu.au; 7 Microelement Research Center, College of Resources and Environment, Huazhong Agricultural University, Wuhan 430070, China; qrajput64@gmail.com; 8 Key Laboratory of Environment Correlative Dietology (Huazhong Agricultural University), Ministry of Education, Wuhan 430070, China

**Keywords:** *Hermetia illucens*, black soldier fly, insect antimicrobial peptides, structure–activity relationship, mechanism of action, antimicrobial resistance, AMP prediction, machine learning, AlphaFold, genomics, transcriptomics, peptide engineering

## Abstract

Antimicrobial resistance (AMR) was associated with 4.95 million deaths in 2019 and may cause 10 million deaths annually by 2050. We synthesize evidence on how the black soldier fly (*Hermetia illucens*) has evolved an expanded antimicrobial peptide (AMP) repertoire, which structural features drive family-specific activity, what mechanisms are directly demonstrated in *H. illucens*, and how AI contributes. PubMed, Web of Science, and Scopus (plus targeted Google Scholar) were searched from inception to 1 February 2026; studies were included when they reported BSF peptide identities, expression/proteomics, evolutionary analyses, quantitative activity, mechanistic assays, or BSF-focused computation, and claims were tiered as predicted, expression-supported, or experimentally supported. The literature supports 50–80 BSF AMP genes, plausibly shaped by gene duplication and balancing/diversifying selection in microbe-rich substrates, with marked induction plasticity across tissues, development, diet, and challenge. SAR is family-dependent: defensin-like peptides rely on disulfide-stabilized CSαβ folds and cationic surface topology; cecropin-like peptides on amphipathic α-helices with selectivity trade-offs; attacin-like peptides on β-architecture where charge-based heuristics are weak; and diptericin/proline-rich peptides remain largely inference-driven in BSF. Mechanistic evidence is strongest for membrane/envelope-centered killing by DLP4 and pore-associated envelope disruption by a recombinant attacin-like peptide, whereas pore geometry, oligomerization, intracellular targets, and broad “resistance-proof” claims remain unresolved. Key gaps include assay heterogeneity, salt/serum stability, selectivity/toxicity, resistance-risk testing, and limited *in vivo* validation, which must be addressed for credible AMR-relevant translation.

## Simple Summary

Antimicrobial resistance is increasing demand for new anti-infectives. This review summarizes what is known about antimicrobial peptides (AMPs) from the black soldier fly (*Hermetia illucens*), focusing on why they are diverse, how they are regulated, which structural features control activity, and what mechanisms are actually proven in BSF. Evidence shows that BSF encodes an expanded AMP repertoire shaped by its microbe-rich ecology and gene duplication, with strong changes in expression across tissues, diet, development, and infection. Defensin-like peptides and cecropin-like peptides show family specific structure–activity patterns, while attacin-like and diptericin/proline-rich peptides remain less resolved. AI tools help prioritize candidates, but predictions often fail without validation of processing, stability, selectivity, and mechanism.

## 1. Introduction

Antimicrobial resistance (AMR), defined as the ability of microorganisms to withstand exposure to antimicrobial agents that were previously effective against them, has become one of the top ten global public health threats. In 2019, AMR was associated with approximately 4.95 million deaths worldwide, and it is estimated that may cause 10 million deaths per year by 2050 [1]. The alarming rise of multidrug-resistant microorganisms highlights the need for the discovery and development of novel antimicrobial agents.

Among the alternatives under investigation, antimicrobial peptides (AMPs) have emerged as highly promising candidates. AMPs are conserved molecules that serve as a first line of defense in plants, microorganisms, and animals [2]. Their broad-spectrum activity, rapid mode of action, and low propensity for inducing resistance make them attractive leads in the search for next-generation therapeutics [3,4]. AMPs are critical components of innate immunity in insects, which lack an adaptive immune system [5]. Insects produce structurally diverse structural AMPs, such as cysteine-rich defensins, amphipathic α-helical cecropins, proline-rich peptides, and glycine-rich attacins, which provide protection against bacteria, fungi, and viruses [6,7]. This diversity, combined with their ecological ubiquity and adaptability, positions insect-derived AMPs as a particularly rich and underexploited resource for antimicrobial discovery. Among insects, the larvae of black soldier fly (here on out will be referred to as BSF) has emerged as a model saprophagous insect with exceptional potential as a source of AMPs [8]. Unlike insects in sheltered or symbiotic niches, which often lose redundant AMP genes to reduce self-toxicity and minimize energetic costs [3], BSF larvae inhabit microbe-rich substrates such as manure and decomposing organic matter [4,9]. This constant pathogen exposure has favored the evolution and diversification of extensive AMPs. Genomic and transcriptomic analyses estimate that BSF genome encodes between 50 and 80 AMP genes, significantly more than many other insects [10]. Advances in high-throughput sequencing and bioinformatics, with tools like Ampir “https://ampir.marine-omics.net/ (accessed on 6 December 2025)”, CAMPR3 “https://camp3.bicnirrh.res.in/ (accessed on 6 December 2025)”, and AlphaFold2 “https://deepmind.google/science/alphafold/ (accessed on 6 December 2025)”, have revolutionized the discovery, annotation, and modelling of BSF-derived AMPs [11,12]. These approaches enable detailed characterization of physicochemical properties (net charge, hydrophobicity, and amphipathicity) and facilitate the optimization of peptide rational design to enhance activity while minimizing host toxicity [13].

Despite their promise, AMPs are not intrinsically “resistance-proof.” Bacteria can adapt through surface charge remodeling (e.g., teichoic acid or lipid A modification), protease secretion, efflux, biofilm-associated tolerance, and stress-response rewiring, and these routes can be selected under chronic or sublethal exposure [14,15,16,17]. Therefore, any AMR-relevant AMP development pipeline should explicitly evaluate resistance risk (serial passage/experimental evolution), cross-resistance potential, and mitigation strategies (combination therapy, multi-target cocktails, and exposure minimization). In parallel, computational prioritization (machine-learning AMP classifiers and structure prediction) accelerates candidate selection but cannot substitute for evidence on peptide processing, post-translational modifications, stability in salt/serum, and selectivity; these factors are frequent causes of false positives and inflated activity estimates. Accordingly, throughout this review we separate BSF-supported mechanisms from inter-species inference and treat in silico outputs as hypotheses that require defined validation steps [18].

Evidence language policy. Throughout this review, we distinguish (a) predicted findings (sequence-based AMP classifiers, structural models, docking), (b) expression-supported findings (challenge/diet/tissue-dependent transcripts or proteomics in *H. illucens* without purified peptide testing), and (c) experimentally supported findings (purified/recombinant/synthetic peptides tested with quantitative activity endpoints, and where available, mechanistic assays and *in vivo* models). Mechanistic assignments from other insects are labelled as inferred unless demonstrated in *H. illucens*.

The central question of this review is which evolutionary and structure–activity principles explain the diversification of BSF AMPs and determine their regulation and antimicrobial mechanisms, and how can these principles be exploited using modern bioinformatics and AI tools to advance BSF AMPs toward applications that mitigate antimicrobial resistance? To address this question, we organize the review around six guiding questions: (i) What is the current evidence-based inventory of BSF AMPs and how can they be classified into defensin, cecropin, attacin, and proline-rich/diptericin families? (ii) Which evolutionary processes (gene duplication, balancing/diversifying selection, and rare acquisition events) plausibly explain their unusually expanded repertoire? (iii) How are BSF AMP genes regulated across tissues and developmental stages, and how do microbial, dietary, and environmental stimuli reshape expression profiles? (iv) Which physicochemical features (net charge, amphipathicity, hydrophobic moment, disulfide-stabilized folds, and sequence motifs) are linked to specific antimicrobial mechanisms (membrane disruption, pore formation, and intracellular targeting), and what evidence is BSF-specific versus inferred from other insects? (v) How reliably do in silico tools (e.g., AMP predictors and structure prediction) prioritize functional candidates and what are their main limitations (false positives, post-translational modifications, folding/stability constraints)? (vi) What bottlenecks currently limit translation (*in vivo* validation, formulation/delivery, manufacturing scalability, and resistance management) and what research priorities most directly address them?

Throughout, we use a strict definition of AMPs as gene-encoded peptides whose primary antimicrobial function arises from peptide physicochemistry rather than enzymatic catalysis; therefore, lysozymes are discussed as synergistic humoral effectors but excluded from AMP counts and AMP SAR/mechanism tables. This framing makes explicit which claims are supported by BSF-specific experimental evidence and which represent mechanistic hypotheses that remain to be tested in *H. illucens*.

## 2. Methodology

A structured literature search was conducted to identify studies on *H. illucens* (BSF) AMPs, encompassing AMP diversity/nomenclature, evolutionary expansion and selection, regulation under microbial/dietary/environmental stimuli, structure–activity relationships (SAR), and mechanisms of action. Searches were performed in PubMed, Web of Science Core Collection, and Scopus, supplemented by targeted Google Scholar queries to capture early-online and grey literature, and were run from database inception to 1 February 2026. Query blocks combined organism terms (“*Hermetia illucens*” OR “black soldier fly” OR BSF) with AMP/family terms (AMP* OR “antimicrobial peptide*” OR defensin* OR cecropin* OR attacin* OR diptericin* OR “proline-rich”) and outcome/context terms (MIC/MBC, mechanism, membrane disruption/permeabilization, “structure–activity,” amphipath*, “hydrophobic moment,” transcriptom*, proteom*, expression, duplication/selection/evolution), with an additional computational block (Ampir, CAMPR3, AlphaFold, I-TASSER “https://aideepmed.com/I-TASSER/ (accessed on 6 December 2025)”, machine learning, structure prediction, docking, molecular dynamics). Studies were included if they (i) defined BSF AMP sequences/identities, (ii) reported quantitative antimicrobial activity for defined peptides (MIC/MBC or concentration-linked inhibition assays), (iii) provided expression/proteomic evidence of AMP induction in BSF, (iv) analyzed duplication/selection of BSF AMP loci, or (v) applied computational pipelines directly to BSF datasets; studies not involving BSF (unless used explicitly as background and labeled as non-BSF inference), lacking sufficient methodological detail, or focusing solely on non-AMP enzymatic antimicrobials were excluded. Titles/abstracts were screened followed by full-text assessment; backward and forward citation-chaining was used to minimize omission of primary studies, and primary experimental sources were prioritized when multiple reports existed. Data extracted included peptide/gene identifiers and mature sequences, family assignment, assay conditions and endpoints, targets/strains, mechanistic readouts, and *in vivo* evidence, and synthesis explicitly separated predicted, expression-supported, and experimentally supported claims, labeling cross-species mechanism assignments as inferred unless demonstrated in BSF; where physicochemical indices were not reported, values (e.g., whole-peptide hydrophobic moment) were computed in this review and clearly identified as author-derived rather than experimental measurements.

## 3. Evolutionary Drivers of BSF AMP Diversity

In insects, AMP genes evolve under a combination of balancing and diversifying selection pressures. Balancing selection maintains multiple alleles within populations, preserving amino-acid polymorphisms that broaden the spectrum of pathogen recognition and defense [4]. In contrast, diversifying selection becomes prominent after gene duplication events, fostering the accumulation of novel mutations and shifting expression patterns. This process facilitates neo-functionalization, enabling AMP variants to target emerging pathogens or adapt to specific ecological niches [19,20]. These dual evolutionary forces -allelic maintenance and divergence- operate in concert to ensure both broad-spectrum defense and rapid adaptability against evolving microbial threats. The co-evolutionary interaction between hosts and pathogens imposes reciprocal selective pressures, wherein pathogens evolve mechanisms to circumvent host immune defenses, prompting hosts to expand and diversify their antimicrobial peptide repertoires in response. While many insects’ immune genes, such as recognition receptors and signaling molecules, exhibit rapid adaptive divergence, early studies observed surprisingly limited divergence in insect AMP genes, suggesting functional redundancy. It was proposed that AMPs act collectively as a cocktail, with no single peptide under strong directional selection [4]. However, more recent research has revealed a more complex scenario, especially in BSF, where AMP loci show signatures of both balancing selection and post-duplication divergence, underscoring their role in broad-spectrum immune defense [3,4,9,21]. The extensive AMP repertoire in BSF reflects its ecological niche, which exposes this insect to diverse and dense microbial communities in decomposing organic matter. This environment has driven recurrent gene duplication and selection for AMP diversity, providing a robust antimicrobial arsenal. Rare events, such as horizontal gene transfer, have further expanded this repertoire; for instance, the acquisition of a Kunitz-domain in a novel defensin-like peptide introduced new antimicrobial traits [12,22].

Genomic analyses have identified three major AMP duplication events in BSF, resulting in 60 defensin and cecropin paralogs [23,24]. Functional specialization among these paralogs is evident in small variations of net charge and hydrophobicity, which modulate their antimicrobial spectra. For example, defensin-like peptide DLP2 and DLP4 differ by just two surface residues, yet this minor divergence shifts their activity spectrum to include Gram-negative bacteria [25].

**Figure 1:**
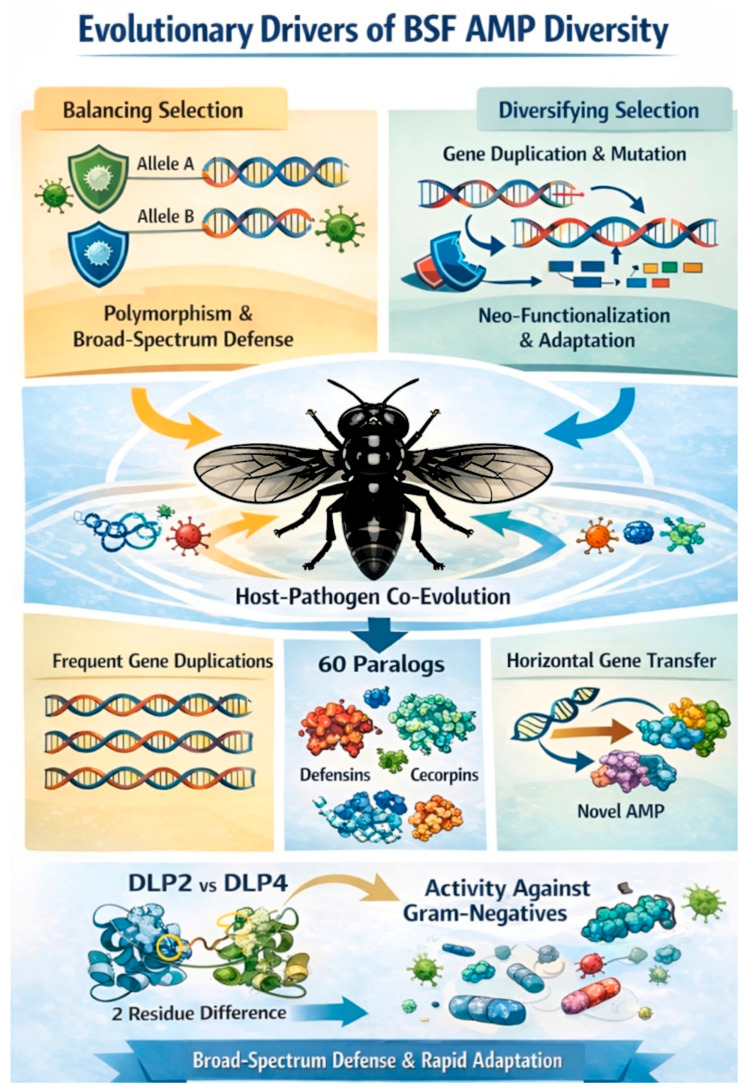
Evolutionary drivers of BSF-AMP diversity. created by a combination of software miro “https://miro.com (accessed on 1 Februray 2026) and pixlr “https://pixlr.com (accessed on 1 Februray 2026)”.

These evolutionary dynamics have produced AMP variants with optimized structural features, thereby enabling the host to rapidly neutralize diverse pathogens and mitigate the ensuing physiological stress. Figure 1 illustrates the evolutionary drivers of BSF antimicrobial peptide diversity.

## 4. Diversity and Classification of BSF AMPs

Insects produce an extensive array of AMPs as a frontline defense against diverse microbial threats. Unlike bacterial bacteriocins, which are often narrow-spectrum and synthesized via non-canonical pathways, insect AMPs are gene-encoded, composed of standard amino acids, and exhibit remarkable diversity even within a single species [26,27]. This molecular diversity equips insects with a versatile antimicrobial arsenal capable of targeting a wide range of pathogens. In the BSF, the AMP repertoire encompasses 50 to 80 genes, categorized into four principal structural classes: (1) defensins, which are 34–51 amino acids long and stabilized by three disulfide bridges forming a CSαβ fold; (2) cecropins, comprising 35–40 amino acids with two amphipathic α-helices; (3) attacins, large glycine-rich peptides of 150–250 amino acids, forming β-sheet structures. In insects, attacins are proposed/known to disrupt Gram-negative outer membranes via lipopolysaccharides (LPS) interactions; and (4) Proline-rich peptides, typically 80–100 amino acid-long, that translocate into bacterial cells to impair intracellular targets. Understanding the diversity of these structural classes is fundamental for elucidating their mechanisms of action. Central to AMP research is the exploration of structure–activity relationships (SAR), as even minor variations in sequence or folding can profoundly influence antimicrobial potency and spectrum. For instance, the net positive charge plays a pivotal role in the initial interaction of the AMPs with the anionic bacterial surfaces as well as the hydrophobic content. Indeed, excessive hydrophobicity leads to aggregation or poor membrane interaction [27,28,29,30,31,32,33,34], while peptide length is index of the concentration needed for having an effective response, i.e., 37–100 aa peptides supports nuanced or dual mechanisms, whereas truncated fragments (<20 aa) require significantly higher concentrations [34,35,36,37,38]. The BSF AMP repertoire offers an ideal model to study these correlations, providing valuable insights for the rational design of next-generation antimicrobial agents aimed at mitigating pathogen-induced stress. In the following paragraphs, we will explore the different structure of the most important classes of AMP in *H. illucens* and their relationship with function.

### 4.1. Defensins (Cysteine-Rich AMPs)

**Figure 2:**
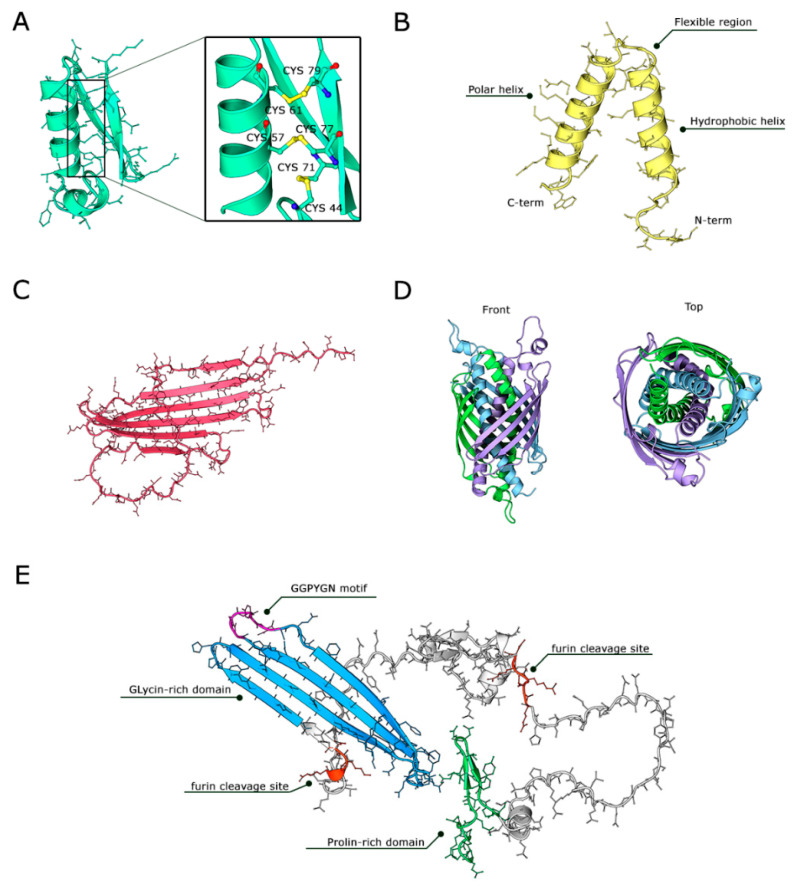
Structural features of the different AMPs classes. (**A**) Defensin-like peptide structure with an α helix and β sheets conformation. Inset: cysteine residues fundamental for the correct folding of the AMP are indicated in yellow. (**B**) Cecropin-like peptide structure with the hydrophobic helix at the N-terminus and a more polar helix at the C-terminus. (**C**) Attacin monomer structure with six anti-parallel beta sheets and the disorder coil of the glycine-rich domain. (**D**) Homotrimer structure of the attacin in a front and top view. (**E**) Diptericin structure with the proline-rich domain in green, the glycine-rich domain in light blue, and the furin cleavage domain and the GGPYN domain in red and purple, respectively. All structures shown are AlphaFold-predicted models generated from amino-acid sequences and visualized for illustrative purposes. These models are not experimentally determined structures, and any inference about oligomerization, pore architecture, or membrane insertion should be treated as a testable hypothesis requiring biophysical/structural validation (e.g., disulfide mapping, membrane-bound spectroscopy, or cryo-EM/NMR) [50].

Defensins are widely conserved across insect orders, including Coleoptera, Diptera, Hymenoptera, and Lepidoptera, where they play a crucial role in innate immunity [31,39]. They are small, cysteine-rich AMPs, typically composed of 34–51 amino acids in their mature form, characterized by six conserved cysteine residues forming three intramolecular disulfide bridges (Figure 2A). These disulfide bonds stabilize αβ (CSαβ) fold, a structural motif comprising an N-terminal loop, a single α-helix, and an antiparallel β-sheet [40]. This conformation is essential for maintaining the peptide’s structural integrity, conferring resistance to proteolytic degradation, and enabling effective interaction with microbial membranes [5,41,42]. BSF encodes multiple defensin isoforms (DLP1–DLP4), synthesized as larger precursors of ~102 amino acids and a mature active form of ~40-residue [43,44]. All of them preserve the canonical cysteine motif and CSαβ fold [45,46]. The structural rigidity imparted by the disulfide-stabilized CSαβ fold not only enhances resistance to proteases, ensuring peptide stability in the hemolymph, but also facilitates precise insertion into bacterial peptidoglycan layers. Mass spectrometry has confirmed the importance of these disulfide bonds, as disrupting any of the three bridges results in complete loss of antimicrobial activity [42]. The cationic nature of defensins, primarily due to lysine and arginine residues, facilitates electrostatic interactions with the negatively charged surfaces of bacterial membranes. BSF defensins, particularly DLP2 and DLP4, exhibit broad-spectrum antimicrobial activity, effectively targeting Gram-positive bacteria such as *Staphylococcus aureus* and methicillin-resistant *S. aureus* (MRSA) both *in vitro* and *in vivo*. Some variants also demonstrate activity against Gram-negative bacteria like *Escherichia coli*, attributed to subtle differences in surface charge distribution that expand their target range [40,43,47]. Additionally, certain insect defensins, including those from BSF, have been shown to inhibit the growth of filamentous fungi, indicating a broader antimicrobial spectrum [41,48,49]. Evidence status (BSF). For DLP2/DLP4, BSF-specific studies provide function-supported evidence (quantitative antibacterial activity of defined peptides) and for DLP4, *in vivo* efficacy has been reported in infection models. By contrast, fine-grained structure–mechanism claims (exact pore geometry, oligomerization state, membrane-bound conformations) remain incompletely validated and should be treated as hypotheses unless supported by direct mechanistic/structural assays.

### 4.2. Cecropins (α-Helical AMPs)

Cecropins are linear, amphipathic peptides typically comprising 35–40 amino acids, characterized by the absence of cysteine residues and the formation of a two-helix α-helical structure [26,27]. In BSF, an expanded cecropin gene family has been identified, with 33 cecropin-like genes [51]. The classical architecture of BSF cecropins consists of a highly hydrophobic N-terminal α-helix, which facilitates membrane insertion, followed by a flexible hinge region and a more polar C-terminal helix that stabilizes interactions with lipid headgroups (Figure 2B). This amphipathic design enables cecropins to effectively integrate into bacterial membranes, is consistent with membrane destabilization through “barrel-stave” or “carpet” models of pore formation (see Section 4). Experimental truncation of cecropin-like peptide 1 (CLP1) demonstrated that the N-terminal helix is critical for its bactericidal activity, consistent with these membrane-lytic mechanisms [7,52]. Subtle sequence variations among cecropin isoforms, such as CLP2 and CLP3, modulate their antimicrobial spectrum by altering surface charge distribution and hydrophobicity. Minor amino acid substitutions can shift target specificity between Gram-negative and Gram-positive bacteria, demonstrating a finely tuned SAR [32]. Indeed, the efficiency of membrane insertion, and thus antibacterial potency, is closely correlated with the hydrophobic moment (μH) of the peptide. Hydrophobic moment and amphipathic charge topology are consistently associated with membrane interaction propensity in α-helical AMPs, but quantitative thresholds (e.g., single μH cutoffs) are rarely transferable across peptides because activity is strongly context-dependent (medium, salt, membrane composition, and peptide aggregation). For α-helical AMPs, amphipathicity (often approximated by μH) is generally associated with membrane interaction propensity. In BSF, some cecropin-like peptides show rapid bactericidal kinetics under defined assay conditions, but μH thresholds are not transferable across peptides and must be treated as design heuristics requiring standardized experimental confirmation [7,18,53]. However, excessive hydrophobicity can increase cytotoxicity toward host cells, underscoring the importance of balancing amphipathicity for selective antimicrobial action [41,54]. Evidence status (BSF). Several BSF cecropin-like peptides have function-supported antibacterial activity *in vitro*, consistent with general α-helical AMP paradigms. However, mechanistic assignments (barrel-stave vs. carpet vs. mixed modes) are typically inferred unless supported by direct membrane assays (depolarization/permeabilization, vesicle leakage, microscopy under defined ionic/serum conditions).

### 4.3. Attacins (Glycine-Rich AMPs)

Attacins are large AMPs, typically ranging from 150 to 250 amino acids, originally identified in moths and now recognized across several insect species, including *H. illucens*. Attacins are typically cysteine-free, enriched in charged and glycine residues, and adopt a characteristic architecture with a flexible N-terminal region and a β-sheet-rich C-terminus. These peptides have attracted considerable interest due to their potent activity against a broad spectrum of Gram-negative bacteria and even some Gram-positive strains [26,27]. In BSF, a 17 kDa attacin was purified from larvae immunized with *E. coli*, displaying significant antimicrobial efficacy against both *E. coli* and MRSA [41,55]. Although, not experimentally resolved, the structural prediction revealed a disordered N-terminus and a well-structured C-terminal region composed of six antiparallel β-strands (Figure 2C). The high concentration in glycine residues, typically 14–22%, suggests an intrinsically disordered conformation in aqueous environments, and a change in conformation upon encountering bacterial membranes or LPS. Sequence-based modeling and structure prediction have proposed β-sheet-rich architectures and possible oligomerization compatible with pore-associated membrane damage. Experimentally, BSF attacin-like peptides have been associated with envelope damage/permeability phenotypes, but the oligomeric state and membrane-bound conformation are not yet established by structural/biophysical methods. Sequence-based modeling and structure prediction suggest a β-sheet-rich architecture and possible oligomerization compatible with pore-associated envelope disruption. In BSF, envelope damage/permeability phenotypes have been reported for attacin-like peptides; however, the oligomeric state and membrane-bound conformation (e.g., β-barrel vs. alternative assemblies) remain unconfirmed by structural/biophysical methods and should be treated as inferred hypotheses (Figure 2D). Sequence integrity is critical, as minor alterations disrupting β-strand alignment or oligomer formation can abolish antimicrobial function [41,56]. Interestingly, the antimicrobial potency of attacins does not correlate directly with conventional physicochemical properties such as net charge, hydrophobicity, or amphipathicity, as seen with smaller AMPs. BSF attacins, which range in net charge from −3 to +10, rely instead on specific sequence motifs and their ability to oligomerize upon membrane contact [57]. Evidence status (BSF). BSF attacin-like peptides have been associated with envelope damage and antibacterial activity consistent with membrane involvement. Structural models proposing β-barrel oligomers are predicted, and require direct confirmation (oligomerization assays, membrane-bound structural/biophysical measurements) before being interpreted as established pore architectures.

### 4.4. Proline-Rich AMPs and Diptericins

Proline-rich AMPs (PRPs) are a distinct class of insect defense peptides with mechanisms that differ from α-helical or β-sheet AMPs. Rather than disrupting membranes directly, PRPs such as drosocin and abaecin adopt flexible, unstructured conformations that enable them to cross bacterial membranes and target intracellular components, particularly in Gram-negative bacteria [31,53,58]. Their moderate positive charge facilitates initial binding but often requires bacterial transport systems (e.g., SbmA) for internalization, which can restrict their spectrum to susceptible species. Proline-rich AMPs in other insects can enter Gram-negative bacteria via transporters such as SbmA and inhibit intracellular processes (ribosomes/chaperones); however, these uptake routes and targets have not yet been experimentally validated for BSF diptericin-like peptides and should be considered inferred hypotheses requiring direct testing in BSF [59,60]. Interestingly, a 95 amino acid proline-rich peptide (HiCG13551) was recently discovered in BSF after bacterial challenge, showing activity against Gram-positive *Staphylococcus* and *Bacillus* species [61].

Diptericins are typically 80–95 amino acids in length, and contain a proline-rich domain (P-domain) and glycine-rich domain, which prevent the formation of stable α-helices or β-sheets in solution, resulting in intrinsically disordered structures [62]. In BSF, 10 diptericin-like genes have been predicted, seven of which are inducible after bacterial challenge [62,63]. It is noteworthy that diptericins from BSF contain only three proline residues within the P-domain, two of which are positioned adjacent to the signal peptide (Figure 2E). This unusual arrangement suggests an association with the dipeptidyl peptidase motif, rather than with classical proline-rich AMPs [64]. Moreover, diptericin retain critical threonine or serine residues that undergo O-linked glycosylation, a modification essential for antibacterial activity, and they also conserve the universal GGPYGN motif previously described in *Drosophila* DptB [64]. Evidence status (BSF). For diptericin-like and proline-rich peptides, much of the proposed intracellular targeting framework derives from other insects; BSF-specific evidence is often limited to expression induction and sequence-based prediction. Direct demonstrations of uptake routes (e.g., SbmA dependence), intracellular targets, and quantitative potency under standardized conditions remain priorities.

### 4.5. Uncharacterized Peptides

Given the massive transcriptomic dataset available but not fully analyzed yet, it is likely that BSF possesses novel AMP families that do not conform to traditional classification schemes. For example, defensin-like peptides containing Kunitz motifs have been identified, which may represent either a unique AMP family specific to BSF or an exceptionally divergent subfamily of defensins [12]. Additionally, some small peptides predicted by in silico screening could be exclusive to BSF, lacking clear homologs in other insects.

## 5. Internal and External Factors Modulating AMP Expression

The capacity of BSF to modulate AMP expression in response to microbial threats and environmental factors reflects an energy-efficient and highly adaptive immune strategy. This ensures precise deployment of AMPs, optimizing defense while conserving metabolic resources. From a biotechnological perspective, leveraging these regulatory pathways, through controlled immune stimulation, dietary interventions, or genetic modulation, offers significant potential for enhancing AMP production in BSF. Such approaches could maximize the yield of specific AMPs for therapeutic and agricultural applications.

### 5.1. Constitutive Expression, Tissue-Specificity, Hormonal and Developmental Regulation

BSF larvae maintain a baseline expression of AMPs as a surveillance mechanism. Transcriptomic and proteomic analyses have shown that defensins, and cecropins are among the most abundantly expressed AMPs together with lysozymes under non-challenged conditions, with defensins being particularly dominant [63,65]. This constitutive expression likely reflects the BSF’s constant exposure to decomposing organic matter, maintaining a state of immune “priming” that facilitates rapid pathogen clearance [66].

The primary tissue for systemic AMP production in BSF is the fat body, where AMPs are synthesized and secreted into the hemolymph [67]. Together with the fat body, hemocytes, freely circulating or sessile immune cells within the hemocoel, also contribute to AMP synthesis as part of the humoral immune response [66,68]. These cells play essential roles in both development and immunity, producing AMPs and lectins that are subsequently released into the hemolymph to provide rapid, systemic antimicrobial protection [6]. In addition to systemic production, certain AMPs, in particular cecropins, are expressed locally in epithelial barriers such as the gut, where they regulate commensal microbiota and provide a frontline defense against ingested pathogens [69]. This dual localization, systemic synthesis in the fat body and hemocytes, coupled with local epithelial expression, enables both immediate site-specific responses and broader organism-wide immunity.

Also, hormonal control plays a pivotal role in modulating AMP expression during BSF development, specifically involving the ecdysone signaling pathway. AMP gene expression patterns shift dramatically during metamorphosis, with a temporary downregulation during pupation followed by reactivation in adults [70,71]. Hormonal fluctuations, as well as environmental stress cues, can fine-tune immune readiness, balancing developmental transitions with immune defense [72,73].

### 5.2. Regulation of AMP Gene Expression in BSF Under Microbial or Fungal Challenge

BSF larvae possess a diverse repertoire of AMPs and can dynamically regulate their expression in response to infection and environmental stimuli. Understanding how AMP gene expression is modulated is crucial to appreciating the BSF’s immune flexibility and to harnessing its antimicrobial potential through controlled interventions. BSF encodes orthologs of the canonical insect NF-κB pathways (*Toll* and *Imd*) and other immune modules, and multiple studies have shown robust, challenge-dependent induction of antimicrobial effectors at the transcript and/or protein level. However, the step-by-step signaling mechanics (e.g., ligand processing, adaptor usage, and NF-κB activation dynamics) are not yet demonstrated experimentally in BSF and remain inferred from other Diptera. We therefore report BSF results as “established,” and label inter-species extrapolations as “inferred.”

### 5.3. Microbial Challenge and Pathogen-Specific AMP Induction

**Figure 3:**
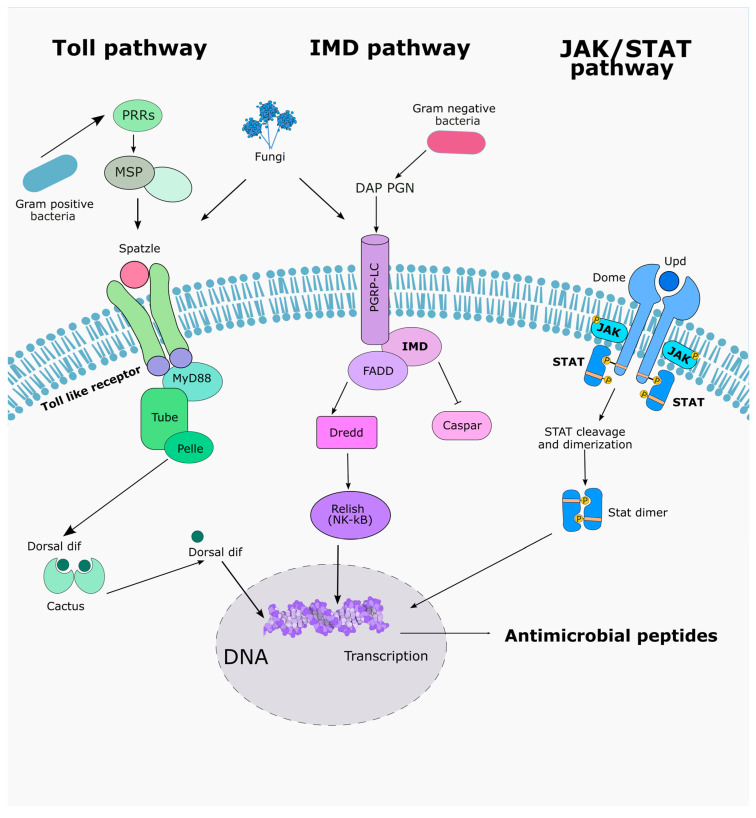
Immune signaling pathways involved in insect immune response against bacteria and fungi. Gram positive bacteria activate the *Toll* pathway (green shades) that triggers Dorsal/Dif release. Gram negative bacteria induce the production of AMPs through the *Imd* pathway (purple shades) that induce the inhibition of Caspar, permitting Dredd-mediated Relish cleavage and nuclear translocation to drive AMP expression. Fungi induce a mixed response that activate both pathways. *JAK/STAT* pathway (blue shades): Unpaired ligands (Upd1-3) bind Dome receptor, causing JAK trans-phosphorylation, STAT cleavage/dimerization, and AMP gene activation. Created with Inkscape 1.3.

Upon microbial challenge, BSF larvae exhibit a rapid and dynamic upregulation of AMP gene expression, orchestrated primarily through the *Toll* and *Imd* signaling pathways, complemented by the JAK/STAT pathway (Figure 3) [24,74,75]. These pathways act as molecular sensors that recognize pathogen-associated molecular patterns (PAMPs) and translate them into transcriptional responses. The *Toll* pathway is predominantly activated by fungal β-glucans and lysine-type peptidoglycans from Gram-positive bacteria. Upon recognition, the serine protease cascade leads to the cleavage of the Spätzle ligand, which binds to *Toll* receptors, initiating a signaling cascade involving MyD88 and Tube that culminates in the nuclear translocation of NF-κB transcription factors Dorsal and Dif. This activation induces the transcription of defensins, attacins, and certain cecropins [67,76,77]. Conversely, the *Imd* pathway is primarily activated by diaminopimelic acid (DAP)-type peptidoglycans found in Gram-negative bacteria. Activation of the *Imd* receptor complex leads to the recruitment of FADD and Dredd, resulting in the cleavage and activation of the NF-κB transcription factor Relish. Activated Relish enters the nucleus and induces the transcription of *IMD*-dependent AMPs, including cecropins, diptericins, and attacins [7,67,78].

Experimental results on immune challenged larvae confirm that *H. illucens* differentially mobilizes its immune repertoire depending on whether the challenge arises from Gram-positive, Gram-negative, or fungal pathogens, yet these responses show significant overlap at the level of AMPs induction. Gram-negative bacteria such as *E. coli* strongly activate the *Imd* pathway through recognition of DAP-type peptidoglycan, leading to the induction of cecropins, attacins, and diptericins [65,67]. Transcriptomic analyses revealed that several cecropin isoforms of cecropin-like-peptide 3 (CLP3) (e.g., LOC119656623, LOC119656627, LOC119656630) are consistently upregulated in response to Gram-negative infections, correlating with rapid bacterial clearance observed in the hemolymph [51]. These peptides are highly effective against Gram-negative targets, consistent with their ability to disrupt bacterial outer membranes. In contrast, Gram-positive bacteria such as *S. aureus* engage the *Toll* pathway through lysine-type peptidoglycan recognition, leading to strong expression of defensins and certain cecropins [62]. Interestingly, while defensins represent the canonical *Toll*-regulated effectors, cecropins are also significantly upregulated after *S. aureus* infection, suggesting a broader overlap between the *Toll* and *Imd* cascades in *H. illucens* than in other Diptera. Metabolomic studies further indicate that *S. aureus* elicits profound metabolic remodelling, with amino acid catabolism and purine metabolism fueling prolonged AMP production [79]. This metabolic cost may explain why Gram-positive infections persist longer in BSF hemolymph compared to Gram-negative ones.

Fungal pathogens elicit a more nuanced response. Early transcriptomic studies suggested that fungi primarily activate the *Toll* pathway, leading to the upregulation of defensins and cecropins, while simultaneously triggering lysosomal and autophagy-related processes [51]. This pattern mirrors, to some extent, the response against Gram-positive bacteria, reinforcing the idea of shared recognition and effector modules in BSF immunity. However, recent work has revealed that fungal modulation of AMP expression is highly species-specific. Combined RNA-seq with LC–MS/MS proteomics to characterize AMP profiles under basal conditions and following exposure to *Saccharomyces cerevisiae*, *Candida tropicalis*, and *Beauveria bassiana*. Defensins were the dominant AMP class observed together with lysozymes, but their regulation varied with fungal type. In fact, both *S. cerevisiae* and *B. bassiana* induced strong lysozyme upregulation, consistent with their known antifungal activity, whereas *C. tropicalis* suppressed lysozyme expression, likely facilitating its survival in the gut. Interestingly, *C. tropicalis* triggered marked upregulation of defensins and cecropins, while *B. bassiana* tended to downregulate them, suggesting that commensal-like yeasts and pathogenic fungi exploit distinct immune modulation strategies [24]. The overlap in AMP induction between *S. cerevisiae* and *B. bassiana* indicates shared immune recognition pathways, yet their divergent effects on defensins and cecropins underscore the complexity of host–fungus interactions [24].

### 5.4. AMP Induction by Probiotics

Beyond pathogenic stimuli, beneficial microorganisms can also modulate the BSF immune system through the same pathways but without pathogenic cost for the larvae [80]. Lee et al. (2020) showed that injection of *Lactobacillus casei* upregulated cecropin 3 and defensin 3 in larval hemolymph, resulting in extracts with strong antimicrobial activity against *Enterococcus faecalis*, *Streptococcus mutans*, *Candida vaginitis*, and MRSA, while sparing beneficial *Lactobacillus* species [81]. Choi et al. (2018) purified novel peptides (Hermetia k20 and k22) from *L. casei*-immunized larvae, which displayed potent activity against *Klebsiella pneumoniae* and *Shigella dysenteriae* without cytotoxicity, supporting their potential as novel antimicrobial drug candidates [82].

### 5.5. Diet and Environmental Modulation

BSF thrives in highly diverse and microbe-rich substrates, from agricultural residues to manure and catering waste, and this ecological flexibility is underpinned by a remarkable immune plasticity. Rather than being expressed constitutively, these AMPs are tightly regulated by both the nutritional composition and microbial complexity of the larval diet, making the diet one of the most powerful modulators of BSF immunity.

Nutrient-rich or microbially diverse diets consistently broaden the spectrum and intensity of AMP responses. Feeding on substrates enriched with proteins, brewer’s grains, chitin, cellulose, or sunflower oil induces distinct AMP expression patterns, with protein- and oil-rich diets often driving the strongest upregulation [63]. Strikingly, these effects can surpass even direct bacterial supplementation, suggesting that specific dietary macronutrients and phytochemicals—such as phytosterols in sunflower oil—act as potent immune modulators. Such diet-dependent transcriptional changes translate into functional outcomes: hemolymph from larvae fed on enriched diets displays stronger inhibitory activity against both Gram-positive and Gram-negative bacteria, confirming that AMP regulation has ecological and applied relevance. Studies on food- and waste-based substrates reinforce this pattern. Larvae reared on organic food waste or municipal waste not only show higher AMP transcript abundance, particularly at prepupal stage, but also maintain extended antibacterial activity in their hemolymph [83,84]. Similarly, when reared on catering waste, the expression of both defensin and cecropin was significantly upregulated in larvae fed on substrates supplemented with bread and dairy products, and their hemolymph retained inhibitory effects on *E. coli* and *Micrococcus yunnanensis* up to 48 h [70]. By contrast, fruit- and vegetable-only diets, with their lower protein content, consistently downregulated AMP gene expression, highlighting the importance of balanced macronutrients for immune competence. Indeed, nutrient balance, particularly protein-to-carbohydrate ratio, emerges as a key determinant of this immune-nutritional interaction. Optimal C:N ratios promote AMP transcription, linking macronutrient availability to immune readiness [85]. However, excess dietary protein can compromise immunity rather than strengthen it. Larvae reared on high-protein diets accumulate uric acid in the hindgut, exhibit delayed and reduced adult emergence, and show markedly lower survival after bacterial infection with *Pseudomonas protegens* compared to larvae on low- or medium-protein diets. Thus, while protein enrichment can enhance AMP expression, chronic excess imposes physiological costs and reduces tolerance to pathogen challenge. Taken together, these findings reveal that BSF immunity is not a fixed trait but a nutritionally tuned system that integrates microbial exposure, substrate composition, and developmental stage. The resulting AMP expression profiles not only enable larvae to cope with pathogen-rich environments but also open applied opportunities: by tailoring rearing substrates, it may thus be possible to boost AMP production for functional feed applications, reducing dependence on antibiotics in livestock and aquaculture [63,66].

## 6. Structure–Activity Relationships (SAR) of BSF AMPs

AMP activity is governed by a small set of interdependent structural determinants charge distribution, amphipathicity/hydrophobic moment, hydrophobicity, length, conformational constraint (e.g., disulfides), and processing/post-translational state whose effects are strongly context dependent (ionic strength, serum binding, proteases, membrane composition). Recent high-impact synthesis of AMP translational biology emphasizes that credible optimization requires linking structural arrangements and design modifications to quantitative function, resistance risk, and deployability rather than relying on sequence-based heuristics alone [86]. Within BSF, SAR claims should therefore be tiered as: BSF-demonstrated (purified/recombinant peptide with quantitative MIC/MBC ± mechanistic readouts) versus inferred (assigned by homology or general AMP paradigms). To compare BSF defensin-like (DLP1–4) and cecropin-like peptides (CLP1–3) on a common SAR basis, we summarize mature sequences, reported MICs (when available), and whole-peptide hydrophobic-moment estimates (μH) in Table 1.

### 6.1. Defensin-like Peptides (DLPs): Disulfide-Stabilized Fold and Surface Charge Topology

BSF defensin-like peptides exemplify SAR dominated by fold constraint and electrostatic topology. The CSαβ scaffold stabilized by three disulfide bonds constrains the peptide into a compact architecture that tolerates proteolytic environments and enables reproducible presentation of cationic surface patches for bacterial envelope interaction (Table 2). In BSF, DLP4 has the strongest function-supported evidence base: quantitative MIC data are reported together with direct comparisons to conventional antibiotics under the same assay system, enabling an interpretable potency claim rather than qualitative labels [88]. SAR-relevant takeaway for DLPs, small substitutions that reshape the surface charge distribution can plausibly shift spectrum; however, mechanistic micro-assignments (exact pore geometry, oligomeric state) should be stated as hypotheses unless directly measured.

### 6.2. Cecropin-like Peptides (CLPs): Amphipathic Helices, Hinge Integrity, and Selectivity Trade-Offs

For α-helical cecropin-like peptides, SAR is primarily governed by amphipathicity (including hydrophobic moment), net cationicity, and helical propensity. The N-terminal helix typically provides the membrane-engaging face, while hinge and C-terminal regions modulate insertion depth and selectivity. The practical design constraint is unavoidable: increasing amphipathicity/hydrophobicity can enhance bacterial killing but frequently increases host–cell toxicity and aggregation risk; therefore, SAR claims should be paired with selectivity readouts when available. Cecropin-like peptides are classical amphipathic α-helical AMPs; their membrane interaction propensity is commonly rationalized using amphipathicity proxies such as μH (Table 1). More broadly, AMP design reviews emphasize that rational optimization must consider both activity and translation-limiting liabilities (stability, resistance, delivery) [86].

### 6.3. Attacin-like Peptides: β-Architecture and Pore-Associated Envelope Damage

Attacin-like peptides represent a distinct group where activity does not map cleanly onto the usual small-AMP descriptors (charge, hydrophobic moment) because the functional unit is a larger β-structured architecture with envelope-targeting effects [54]. In BSF, the attacin Hill_BB_C10074 has BSF-specific experimental support for antibacterial activity against *P. aeruginosa* and membrane/envelope damage phenotypes, including electrochemical impedance spectroscopy consistent with pore-associated disruption [54]. SAR implication: sequence/structure features that preserve β-architecture and membrane-interacting motifs are likely more predictive than “more positive charge” rules [54].

### 6.4. Diptericin-like/Proline-Rich Peptides: SAR Is Likely Processing- and Uptake-Dependent (Mostly Inferred in BSF)

In other insects, proline-rich AMPs (PrAMPs) commonly rely on transport-mediated uptake (often involving inner-membrane transporters such as SbmA) and then inhibit intracellular targets (classically translation machinery and/or chaperone systems), so SAR depends on motifs that preserve uptake/target engagement rather than membrane amphipathicity [89,90,91]. However, for BSF diptericin-like/proline-rich candidates, this remains largely expression- or homology-inferred, because the literature still emphasizes gene induction after challenge/injury rather than direct demonstrations of uptake route, intracellular target binding, and standardized MIC/MBC using purified/recombinant peptides [92]. Until those BSF-specific mechanistic steps are shown, SAR statements about “uptake-dependent intracellular targeting” should be framed as testable predictions, not established BSF mechanisms [66].

### 6.5. Why SAR Looks “Inconsistent” Across Studies: Minimum Reporting Needed for Interpretable Comparisons

Across BSF AMP papers, reported potency is frequently non-comparable because MIC/MBC outcomes shift with media composition, ionic strength, inoculum, peptide adsorption/aggregation, endpoint definition, and serum/protease inactivation. Therefore, SAR comparisons should only be made when studies report (at minimum) peptide identity/purity and quantitative MIC/MBC with explicit assay conditions, plus at least one stability or mechanism-adjacent control under physiologically relevant constraints (e.g., salt/serum). Table 2. Illustrates key AMPs from *Hermetia illucens* and their properties. Without this, between-study “SAR differences” are often assay artefacts rather than biology.

**Table 2:** Key AMPs from *Hermetia illucens* and their properties.

Peptide (Family)	Key Motifs	Net Charge	Target Spectrum	Predicted Mechanism	References
Defensin	3 disulfide bridges (CSαβ fold)	+3 to +4 (cationic)	Gram-positive *S. aureus* (including MRSA), some Gram-negatives	Membrane permeabilization/envelope disruption (BSF-supported), specific pore model and intracellular targets (inferred unless directly tested).	[25,43,93]
Cecropin	amphipathic α-helices (N-terminal helix critical)	+6 (highly cationic)	Gram-negative *E. coli* (ampicillin) and *S. aureus*	Membrane disruption (BSF-supported activity, mechanism generally consistent with α-helical AMPs), specific pore model (inferred).	[32,51,94]
Attacin	glycine-rich; β-sheet domains	0 (slightly cationic or neutral)	Gram-negative (*E. coli*, *P. aeruginosa*), also inhibits MRSA growth	Envelope disruption consistent with pore-associated activity (BSF evidence); specific β-barrel architecture remains inferred/predicted.	[41,56,57]
Diptericin	7 paralogs in a Chr2 tandem cluster; HiDpt1–4 functional (P-domain + G-domain), HipDptA–C pseudogenized; 1–2 furin sites (R-X-R/K-R) between domains	Not Reported in BSF (paralog specific)	Gram-negative inducible (10^3^–10^4^×) response to Gram-positive (*M. luteus*)	BSF mechanism not defined; glycine-rich G-domain is the putative active AMP released by furin cleavage.	[62]

Note: All listed peptides are originally derived from BSF or resulted from microbial challenge. Net charges are estimated at physiological pH based on sequence (basic minus acidic residues). Mechanisms are categorized as demonstrated in BSF only when supported by direct mechanistic assays with defined peptides; otherwise, mechanisms are inferred from broader AMP literature and are presented as hypotheses.

## 7. Mechanisms of Action of BSF AMPs

BSF AMPs employ different strategies to neutralize microorganisms, broadly categorized into lytic and non-lytic mechanisms. The firsts involve direct membrane disruption leading to a rapid clearance of the bacteria, while the non-lytic mechanisms rely on intracellular targeting of chaperones, DNA, or RNA after membrane translocation (Figure 2) [27,28,39,95]. Additionally, BSF AMPs often act synergistically, enhancing antimicrobial efficacy through combined actions [67]. Mechanistic models summarized below integrate direct evidence from *H. illucens* and mechanistic paradigms established in other insects where BSF-specific validation is incomplete. We explicitly tag non-BSF literature to avoid over-generalization and to highlight testable predictions for BSF. To avoid mechanistic overreach, Table 3 separates mechanisms directly demonstrated using defined *H. illucens* peptides from mechanisms that remain inferred from other insects.

**Table 3:** Mechanistic evidence matrix for BSF antimicrobial peptides (BSF-demonstrated vs. inferred).

BSF Peptide (Family)	Evidence (*In Vitro/In Vivo*)	BSF-Demonstrated Mechanism (Direct Readout)	Still Inferred/Not Demonstrated in BSF	References
DLP4 (defensin-like)	*In vitro* (*S. hyicus*, antibiotic comparators), *in vivo* (mouse abscess), serial passage	Envelope/cell-wall damage phenotypes consistent with membrane-centered killing, *in vivo* efficacy, no MIC increase after repeated passages under selection (study conditions)	Specific pore model, oligomerization, intracellular targets	[88]
DLP2, DLP4 (defensin-like)	*In vitro* (MDR *S. aureus*), *in vivo* (mouse protection)	Antibacterial activity with measured damage/functional mechanistic readouts under study conditions, *in vivo* protection	Exact pore architecture, general “resistance-proof” claims beyond tested strains/conditions	[43]
DLP4 (defensin-like)	*In vitro* (*C. difficile* clinical isolates)	Direct activity with microscopy-reported envelope damage consistent with membrane/cell-wall disruption	Spectrum generalization, pore architecture, intracellular targets	[96]
Hill_BB_C10074 (attacin-like)	*In vitro* (recombinant, *P. aeruginosa*)	TEM envelope damage + impedance spectroscopy consistent with pore-associated permeabilization	Predicted β-barrel/homotrimer architecture, membrane-bound structural state	[54]

*Note:* Mechanisms are classified as “BSF-demonstrated” only when supported by direct mechanistic readouts using a defined BSF peptide (purified/recombinant/synthetic) in the cited paper; otherwise statements are inferred and must be labelled as such.

### 7.1. Lytic Mechanisms: Membrane Disruption and Pore Formation

**Figure 4:**
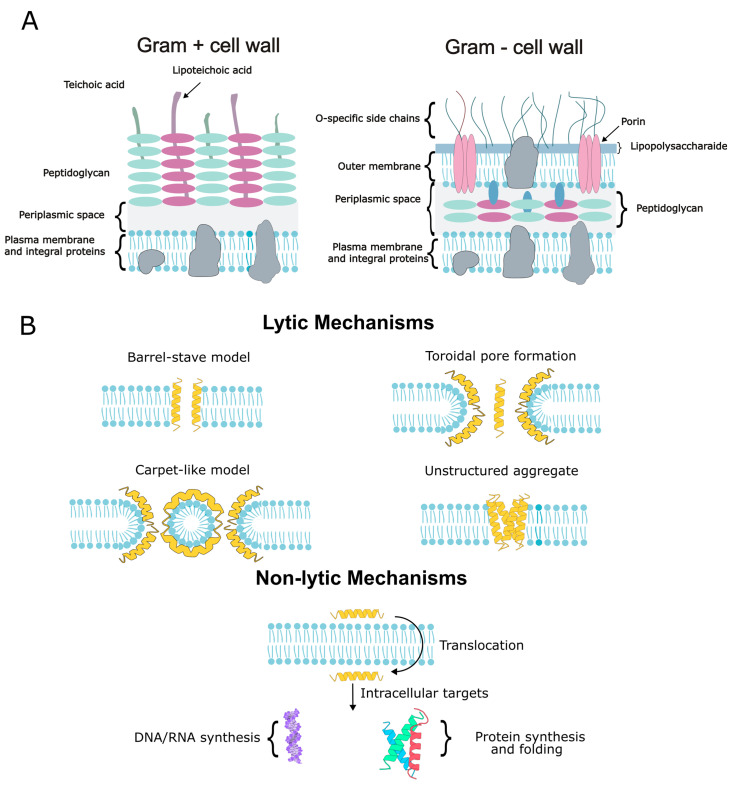
Mechanism of action of antimicrobial peptides on bacterial cells. (**A**) Structure of Gram-positive and Gram-negative bacterial cell wall. (**B**) Non-lytic mechanism of action of antimicrobial peptides: barrel-stave model, toroidal model, carpet-like model, and unstructured ring pores. Non-lytic mechanisms of action of AMPs, mediated by interactions with proteins, DNA, and RNA. Created using Inkscape 1.3.

The primary mode of action for many BSF AMPs involves the disruption of microbial membranes through electrostatic interactions with negatively charged surface molecules such as LPS in Gram-negative bacteria or teichoic acids in Gram-positives (Figure 4A).

This initial binding enables peptides to destabilize membranes via several distinct structural models. The following pore/lysis models are established frameworks from broader AMP literature unless explicitly indicated as BSF-demonstrated, their application to specific BSF peptides should be interpreted as inferred (Figure 4B). In the barrel-stave framework, peptides can form channels that facilitate ion leakage and membrane depolarization, leading to cell death [32,51]. Defensin-like peptides, including DLP4, have been reported to disrupt bacterial envelope integrity and increase membrane permeability in BSF-specific studies; however, the specific pore architecture (toroidal vs. barrel-stave vs. other models) has not been directly resolved for BSF defensins and remains a mechanistic hypothesis informed by broader AMP paradigms [94,97]. Both mechanisms lead to rapid bacterial death and reduced pathogen persistence. Another prevalent lytic mechanism is the carpet-like model, in which peptides adsorb onto the membrane surface without forming distinct pores. Upon reaching a critical surface concentration, the lipid bilayer collapses into micellar fragments, resulting in detergent-like lysis [27,46]. Applied at high concentrations, BSF cecropins have demonstrated the ability to completely dissolve *E. coli* membranes, consistent with this carpet-like mode of action [7,70,98]. Furthermore, in BSF, shorter AMPs or those targeting highly anionic membranes such as sarcotoxin or stomoxyn analogs (39-residue helices) are prime candidates for this mode of action [99,100]. At very high peptide-to-lipid ratio, peptides can induce unstructured aggregate-driven membrane collapse. In this model, peptides and lipids form disordered micelles, tearing apart the membrane rather than creating defined pores [28,101,102]. Although this mechanism has not been explicitly documented for BSF AMPs, it represents a possible outcome under conditions of peptide over application. Smaller BSF AMPs, if overexpressed, could trigger such generalized membrane fracturing, while larger peptides like attacins are more likely to form structured pores.

BSF-specific mechanistic evidence is strongest for DLP4, where envelope disruption phenotypes and therapeutic effects have been evaluated beyond generic model descriptions. In a pharmacodynamics-focused study, DLP4 displayed quantitative antibacterial activity and produced ultrastructural damage consistent with cell-envelope disruption; importantly, efficacy was also reported in an *in vivo* abscess model, and synergy with conventional antibiotics was explored rather than assumed [88]. These data provide a higher evidentiary standard than model-only assignments (barrel-stave/toroidal/carpet), and they should be used as benchmark examples for how future BSF AMP studies can link SAR to mechanism and to *in vivo* relevance.

### 7.2. Non-Lytic Mechanisms: Intracellular Targets

While membrane disruption is the predominant mode of action, certain BSF AMPs operate through non-lytic mechanisms by translocating into bacterial cells to target intracellular processes (Figure 4B). Proline-rich peptides and defensin-like peptides exemplify these mechanisms. Indeed, they can enter the microbial membranes (via transient pores or transport systems) and target intracellular processes [103,104]. Proline-rich AMPs in other insects can enter Gram-negative bacteria via transporters such as SbmA and inhibit intracellular processes (ribosomes/chaperones); however, these uptake routes and targets have not yet been experimentally validated for BSF diptericin-like peptides and should be considered inferred hypotheses requiring direct testing in *H. illucens* [62]. In other insects, defensins have been reported to interact with intracellular partners (e.g., heat-shock proteins) and can exert bacteriostatic effects; for BSF defensins, this remains to be demonstrated and is therefore treated as an inferred hypothesis [105,106]. Such interactions can be associated with bacteriostatic effects in some systems, buying time for the host immune system to clear the infection. Although attacins are often associated with membrane activity, they also exhibit intracellular effects. Non-lytic effects have been proposed for attacins in other insects (e.g., interactions with envelope proteins/cell wall processes), but for BSF attacin-like peptides the direct evidence currently centers on envelope damage/permeability phenotypes, while intracellular or specific protein-target effects remain inferred. Attacins can oligomerize into β-barrel trimers that integrate into the outer membrane, causing destabilization. Attacins may further inhibit bacterial function by interacting with surface proteins like OmpA or interfering with cell wall synthesis, providing a non-lytic avenue for antimicrobial action [54,56,107].

### 7.3. Synergy and Combined Action

Synergy is a plausible and frequently proposed design principle for insect AMP repertoires, but direct BSF-specific quantification of synergy (e.g., checkerboard/FIC indices, time–kill synergy) remains limited. Where combination effects have been reported (e.g., DLP4 with conventional antibiotics), these findings should be interpreted as study- and condition-specific until replicated across strains and standardized workflows. For example, membrane-disrupting peptides (e.g., attacins or cecropins) can permeabilize the bacterial outer membrane, facilitating the action of enzymes like lysozyme to degrade peptidoglycan. Moreover, PRPs may synergize with membrane-active AMPs, where the latter compromise membrane integrity, facilitating PRP uptake and amplifying antimicrobial efficacy. AMPs can be administered in sublethal concentrations to sensitize bacteria to other antibiotics that act against resistant strains [30]. For instance, early studies suggest that combining DLP4 with oxacillin or vancomycin improve MRSA clearance [7]. Interestingly, BSF cecropins can inhibit *Pseudomonas aeruginosa* biofilm formation and co-administration with biofilm-disrupting agents may create a viable solution for the eradication of established biofilms [28].

### 7.4. Strength of Evidence in BSF: Direct Demonstration Versus Inter-Species Inference

A recurring weakness in the BSF AMP literature is the implicit transfer of mechanistic conclusions from other insects (especially Drosophila) to *H. illucens* without direct confirmation. To avoid mechanistic overreach, evidence can be usefully graded into: (i) direct BSF evidence (purified/recombinant peptide tested on defined strains with quantitative endpoints and mechanistic readouts), (ii) BSF-correlated evidence (challenge-dependent expression changes coupled to crude extract activity), and (iii) inferred evidence (mechanisms assigned primarily by homology or general AMP paradigms). Under this framework, DLP4 has unusually strong BSF-specific support: beyond *in vitro* MIC data, mechanistic effects on bacterial envelope integrity and *in vivo* efficacy have been reported in infection models [88]. Similarly, an attacin-like peptide has been associated with rapid membrane/envelope damage patterns consistent with pore-associated activity, aligning with structural predictions, although rigorous structure–function mapping across membrane compositions and physiological conditions remains limited [54]. In contrast, intracellular targeting by BSF proline-rich/diptericin-like peptides, pathway-to-effector causality (*Toll*/*Imd*/*JAK–STAT* → specific AMP genes), and synergy as a dominant functional principle in BSF are still largely hypotheses supported indirectly or inferred from other Diptera and should be presented as testable predictions rather than established conclusions. This distinction is essential for a translational narrative because mechanistic uncertainty directly affects engineering choices (e.g., amphipathicity tuning versus protein-binding optimization), formulation strategies, and resistance-mitigation design.

### 7.5. Methodological Heterogeneity Inflates Between-Study Variability: Minimum Reporting Standards

A major barrier to critical comparison across BSF AMP studies is inconsistencies in antimicrobial susceptibility testing. Reported potency can shift substantially with changes in medium composition, salt concentration, inoculum size, growth phase, endpoint definition (MIC versus MBC), peptide handling (adsorption, aggregation), and whether serum/protease challenges are included. General AMP susceptibility testing guidance emphasizes standardized broth/agar dilution workflows, explicit reporting of inoculum and endpoints, and inclusion of controls that detect assay artefacts and physiological inactivation [18]. For BSF AMPs specifically, comparability is further weakened by: (i) heterogeneous strain panels (often single laboratory strains), (ii) limited replication and missing confidence intervals, (iii) inconsistent units (µM vs. µg/mL vs. mM without molecular-weight normalization), and (iv) frequent absence of selectivity/toxicity readouts (hemolysis, mammalian cell viability), which is essential when amphipathicity is engineered upward. We recommend, as a minimum reporting set for BSF AMP papers: peptide identity and purity; sequence plus post-translational state; solvent and storage conditions; MIC and MBC with standardized inoculum and media; salt/serum stability tests; time–kill kinetics; and at least one orthogonal mechanistic readout (e.g., membrane depolarization/permeabilization assays and microscopy). Without these elements, differences in “activity” across BSF peptides and families cannot be interpreted as true SAR-driven effects.

### 7.6. Resistance Risk and Adaptation Under Sublethal Exposure (BSF-Specific Evidence vs. Inference)

Despite frequent claims that AMPs are “resistance-proof,” bacterial adaptation can occur, particularly under repeated or sublethal exposure; therefore, resistance risk should be treated as an empirical question rather than an assumption [88]. At present, BSF-specific resistance data are sparse. The strongest direct evidence we identified is for defensin DLP4, where a defined serial-passaging (“resistance induction”) protocol was performed: following 30 sequential passages under peptide selection, the MIC of DLP4 against *Staphylococcus hyicus* did not increase, whereas the antibiotic ceftriaxone displayed an 2-fold MIC increase under the same framework [88]. This finding is consistent with a lower propensity for resistance development under those specific conditions; however, it does not establish resistance-proofing across pathogens, peptides, dosing regimens, or physiological matrices.

For other BSF AMPs (including membrane-active families such as attacins and cecropins), published studies largely emphasize activity and mechanism (e.g., envelope damage and pore-associated effects) but typically do not include standardized resistance-selection experiments, cross-resistance mapping, or genetic/phenotypic characterization of adapted mutants [88]. Consequently, for clinically relevant pathogens frequently used as benchmarks (e.g., *S. aureus* and *P. aeruginosa*), the capacity to evolve reduced susceptibility specifically to BSF AMPs and by which routes remain an open evidence gap.

Recommended minimum for BSF AMP translational claims is therefore: (i) standardized serial passage/experimental evolution with multiple strains and clinically relevant media; (ii) tracking MIC/MBC shifts and kill-kinetics changes (tolerance); (iii) testing cross-resistance to host-defense peptides and to antibiotics; and (iv) mitigation-by-design using justified strategies (e.g., combination therapy, peptide cocktails, exposure minimization) rather than asserting resistance resilience by default [88].

## 8. Integrating In Silico and Experimental Data in AMP Discovery

Computational pipelines (sequence-based AMP classifiers, structure prediction, and docking) are now central to prioritizing candidates from the expanding *H. illucens* genomic and transcriptomic space. However, throughout this review we treat in silico outputs as hypothesis-generating evidence useful for ranking candidates and proposing structural/mechanistic models rather than as validation of antimicrobial function or mechanism. In particular, AlphaFold-predicted structures do not constitute structural proof, and classifier scores (e.g., CAMPR3, Ampir) do not establish that a peptide is expressed, correctly processed, stable, or antimicrobial under physiological conditions. Claims that depend on prediction alone are therefore explicitly labelled as predicted and separated from experimentally supported conclusions [108,109,110,111,112,113]. Table 4 reports examples of in silico predictions used to prioritize BSF AMP candidates or propose mechanisms, alongside the specific experimental observations that were subsequently reported. Computational outputs (classifier scores, AlphaFold models, docking) are hypothesis-generating and are not treated as validation unless paired with purified/recombinant peptide activity and mechanistic assays.

AlphaFold2/3 can produce highly informative atomic models for many folded proteins, but important caveats apply for AMP biology AMPs often involve short length, disorder-to-order transitions on membranes, oligomerization, and context-dependent conformations, all of which may reduce reliability of single-structure predictions and limit mechanistic inference. Accordingly, predicted models are best viewed as starting points to guide experimental design (e.g., mutagenesis, membrane-binding/lysis assays, disulfide mapping), not as stand-alone evidence of pore formation or intracellular targeting [12,34,37,110,111,112,113,116,117,118].

### 8.1. Evidence Levels for Computational Claims in BSF AMP Research

To avoid overstating computational findings, we distinguish five evidence levels for BSF AMPs: Level 1 (Predicted-only): AMP-likeness inferred from sequence features/classifier score; no expression or activity data. Level 2 (Expression supported): transcript/protein detection in tissue/hemolymph after challenge, but no purified/recombinant peptide activity testing. Level 3 (Function supported): purified/recombinant/synthetic peptide shows antimicrobial activity (MIC/MBC or equivalent quantitative assay) under defined conditions. Level 4 (Mechanism supported): direct mechanistic assays (e.g., membrane permeabilization, lipid vesicle leakage, microscopy with membrane damage markers, intracellular target assays) link structure/physicochemistry to killing mode. Level 5 (Structure-supported): experimental structure (NMR/X-ray/cryo-EM) and/or rigorous biophysical validation (disulfide connectivity, oligomerization state, membrane-bound conformation), enabling strong structure–mechanism claims. AlphaFold models, docking poses, and ML scores primarily contribute to Levels 1–2 unless paired with Level 3–5 experiments [12,34,37,110,111,112,113,116,117,118].

Knowing the 3D structure of an AMP can aid in understanding its mechanism of action and the design of analogs. While traditional methods like NMR or crystallography are slow, AI-based tools, such as AlphaFold2, enable rapid prediction of three-dimensional structure with high-quality models [119,120,121]. For example, defensin-like peptides Jg7197, Jg7902, and Jg7904, adopt the canonical αβ-defensin fold with cysteines forming spatially proximal pairs, suggesting a shared mechanism of action despite sequence divergence [12]. Similarly, AlphaFold2 predicted a β-sheet-rich assembly compatible with a pore-associated model. Experimentally, recombinant attacin showed envelope damage by TEM and impedance signatures consistent with pore-associated disruption; however, these data do not establish the specific oligomeric β-barrel architecture, which remains inferred [54,120]. Homology modelling and docking further revealed functional features, such a surface-exposed cluster of positively charged residues in DPL4 that preferentially bind negatively charged bacterial membranes, consistent with experimental vesicles-binding assays [94]. These integrative approaches exemplify how high-quality structural models can guide mechanistic understating and rational peptide engineering. Computational analyses further provide quantitative biophysical indices to predict AMP functionality. For example, the Boman index estimates the propensity of the peptide to bind proteins rather than membranes. Elevated values indicate a higher potential for interacting with intracellular enzyme or receptor, whereas lower values suggest a preference for membrane interactions [122,123]. High Boman index suggests protein-binding propensity; BSF defensins typically exhibit moderate Boman indices, while cecropins, that display lower indices, favor the “carpet” mechanism interaction with membranes. Other parameters, such as hydrophobic ratio, net charge, and hydrophobic moment, correlate with antimicrobial potency [32]. In contrast, peptides predicted to possess antifungal activity (e.g., drosomycin-like sequences) tend to be more polar and to adopt more globular conformations, facilitating insertion into fungal membranes or cell-wall interactions [32]. Predicted candidates are prioritized here by biological relevance support from prediction-only inference (Table 5). Together, these metrics enable AMP rational design: tune net charge, hydrophobic content, hydrophobic moment (amphipathicity), and secondary-structure propensity to maximize target activity, while constraining hydrophobicity to limit host–cell toxicity. These insights have shifted AMP development from single-factor modifications to comprehensive *de novo* design [124].

Despite these advances, computational predictions have limitations. Some candidates may be false positives, failing to be expressed or active *in vivo*, and factors such as folding, stability, and post-translational modifications critical for activity remain challenging to predict [34]. Large-scale testing confirms that computationally prioritized candidates include a non-trivial fraction of experimental failures, arising from both biological and technical causes. In the most comprehensive synthetic screen currently available (36 BSF peptides), one candidate (Hill-Stom2) could not be evaluated due to poor solubility, and at least one cecropin-like peptide (Hill-Cec6) showed no antibacterial activity in the assay panel [28]. Hill-Cec6 failure was attributed to loss of canonical cecropin sequence features (absence of an N-terminal Trp, absence of Pro residues typically present toward the C-terminus) together with reduced cationicity (net charge ~+2) and increased acidic content—changes consistent with impaired amphipathic membrane engagement under the tested conditions [28]. Quantitatively, this context matters because the BSF genome is predicted to encode >50–80 putative AMP genes, yet the literature contains functional *in vitro* validation for only a minority (e.g., 14 peptides were explicitly noted as having published *in vitro* activity prior to that broad screen), meaning that most candidates remain prediction-/expression-supported rather than function-supported [28]. Accordingly, AlphaFold models, classifier scores, and docking should be interpreted as prioritization tools until paired with standardized MIC/MBC testing (including salt/serum stability), and failures (insolubility, incorrect processing/PTMs, low effective cationicity, aggregation, or context-dependent inactivation) should be reported explicitly to calibrate computational pipelines. Moreover, machine learning tools can introduce biases, overpredicting AMP-like sequences in cationic cytosolic proteins [125,126]. These considerations underscore the necessity of experimental validation to confirm predicted functions.

Limitations and risk of over-interpretation. Although in silico tools can markedly accelerate BSF AMP discovery, they introduce predictable failure modes that must be explicitly controlled. First, structure prediction is not structure validation: AlphaFold2/3 outputs represent single (often soluble) conformations and may not capture AMP disorder-to-order transitions, membrane-bound states, oligomeric pores, or alternative folds. Confidence metrics can highlight uncertain regions, but high confidence does not substitute for experimental confirmation, particularly for mechanism claims [121,122,123]. Second, AMP classifier scores are not proof of antimicrobial function. Predictors can overcall AMP-likeness due to compositional bias (cationic residues), dataset leakage, and the well-documented difficulty of constructing unbiased negative sets; consequently, a non-trivial fraction of predicted AMPs fail experimental testing [37,110,111,112,118,127]. Third, docking and scoring are hypothesis-generating: docking can propose plausible peptide–target poses, but scoring functions are approximate and do not establish binding affinity, specificity, or physiological relevance without direct binding/functional assays [128]. For these reasons, we treat computational results as candidate-prioritization tools and emphasize that translation requires standardized expression/processing verification, quantitative MIC/MBC testing under physiologically relevant conditions, and direct mechanism experiments.

### 8.2. Practical Steps to Reduce Computational False Positives in BSF AMP Discovery

Computational AMP pipelines can be made substantially more reliable by treating “AMP-likeness” as a *multi-gate decision* rather than a single classifier score. The following practices reduce false positives at low cost and improve the interpretability of downstream experimental failures. Computational false positives in BSF AMP discovery can be reduced by treating “AMP-likeness” as a staged decision rather than a single score. First, enforce secretion and processing plausibility (signal peptide, propeptide cleavage logic, defensin cysteine motifs) and run predictors on the predicted mature peptide, not the precursor. Second, use ensemble prediction (≥2 classifiers) with disagreement flags, and calibrate ranking against BSF function-supported positives, reporting precision-at-K rather than interpreting scores as probabilities. Third, avoid inflated performance by using homology-aware splits (cluster/family-based) when evaluating models. Fourth, apply “anti-filters” to remove common confounders (low-complexity regions, cytosolic fragments, enzyme domains, long transmembrane helices). Fifth, screen early for solubility/aggregation/adsorption risk and prioritize candidates likely to retain activity under salt/serum conditions. Finally, treat docking and AlphaFold models as hypothesis tools unless paired with standardized MIC/MBC and mechanistic assays, and report inactive/untestable cases to refine future pipelines.

**Figure 5:**
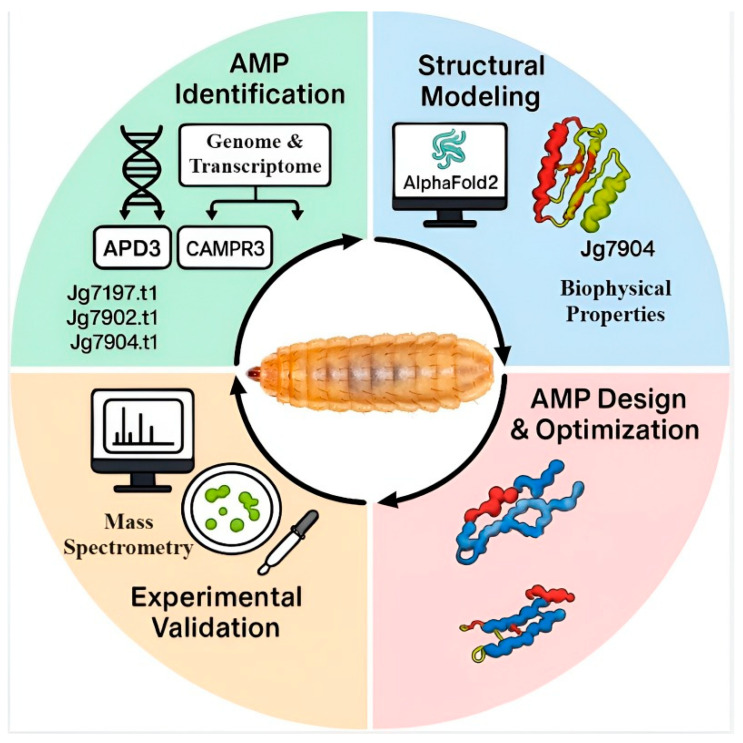
Prediction of AMPs.

The integration with public databases, such as of NCBI “https://www.ncbi.nlm.nih.gov/ (accessed on 1 Februray 2026)”, UniProt “https://www.uniprot.org/ (accessed on 1 Februray 2026), and APD3 can help counteract potential biases related to machine learning or AI-based predictions [129,130]. By cross-referencing novel sequences against experimentally validated entries, researchers can verify motifs, structural features, and functional annotations, reducing the likelihood of false positives. For example, the sequence of BSF DLP4, available in UniProt (ID A0A0M3YE13), annotated with its cysteine motif and literature references, was found to be ~85% identical to *Hi* DLP2 and likely arose from gene duplication. Thus, database integration provides a reliable framework to organize discoveries and inform functional and evolutionary interpretations, complementing computational predictions with experimentally grounded validation. Figure 5 illustrates the workflow on how to predict AMPs.

Future efforts are expected to further integrate computational and experimental approaches. Using molecular dynamics (MD) simulations, for instance, we can predict how different BSF AMPs interact with bacterial versus host membranes to assess selectivity, with predictions subsequently validated through hemolysis assays to ensure minimal toxicity towards mammalian cells. Moreover, in silico tools for optimizing peptides, such as automated design algorithms, can suggest modifications (e.g., end capping and cyclization) to enhance stability, which can then be tested synthetically. Additionally, AI-driven models could predict synergistic AMP combinations. Since BSF produces a cocktail of AMPs in response to infection, computational analyses could potentially identify combinations with the highest synergistic antimicrobial effects, thereby guiding combination therapy studies.

BSF AMP research showcases the seamless integration of bioinformatics and bench science high-throughput approaches. Tools such as Ampir, CRAN “https://cran.r-project.org/package=ampir (accessed on 1 Februray 2026)”, AlphaFold2, AlphaFold Protein Structure Database “https://alphafold.ebi.ac.uk/ (accessed on 1 Februray 2026)”, CAMPR3, and APD3 “https://aps.unmc.edu/database (accessed on 1 Februray 2026)” can rapidly flag numerous peptide candidates from the *H. illucens* genome, while targeted experiments can confirm structural features and mechanisms (e.g., the formation of the attacin pore). This pipeline can not only validate computational predictions in a non-model insect but also sets the stage for designing and deploying optimized AMPs provided challenges in production, delivery, and *in vivo* efficacy are addressed.

## 9. Future Directions and Challenges

Future BSF AMP research should be prioritized around questions that directly resolve current inferential gaps and reduce the distance between *in vitro* potency and deployable efficacy. First, causal genetics is essential RNAi or CRISPR perturbation of pathway nodes (*Toll/Imd/JAK–STAT* components) and candidate AMP loci is required to move from correlation (“induced after challenge”) to causation (“required for clearance”) and to quantify redundancy versus specialization within expanded gene families. Second, mechanistic validation must become conditional- and context-aware. Many peptides that appear potent in low-salt media lose activity in physiologically relevant ionic strength or in the presence of serum proteins and proteases; therefore, susceptibility testing should routinely include salt/serum challenges and orthogonal mechanism readouts (membrane depolarization/permeabilization, microscopy, and time–kill kinetics) under standardized workflows. Third, resistance risk must be treated as an experimental variable, not a slogan. Serial passage experiments, tolerance/biofilm assays, and cross-resistance testing should accompany development-stage peptides, and mitigation strategies (cocktail design, antibiotic combination, and avoidance of chronic sublethal exposure) should be justified with data rather than assumed. Fourth, translation requires selectivity constraints: increasing amphipathicity can increase bacterial killing but also increases hemolysis and mammalian cytotoxicity; thus, every SAR-driven optimization should be paired with selectivity assays and, where possible, pharmacokinetic/pharmacodynamic considerations anchored in BSF-specific studies that already include *in vivo* efficacy (e.g., DLP4) [88,130]. These functions could open avenues in veterinary medicine, vector-borne disease management, or antimalarial drug development.

Scaling production for practical use presents additional challenges. While BSF larvae are inexpensive to rear, direct peptide extraction from the insect biomass is inefficient. Recombinant expression in bacteria, yeast, or insect, or plant systems offer higher yields, though toxicity to host expression systems often requires fusion proteins or secretion strategies. Genetically modified larvae overexpressing specific AMPs could provide a sustainable, high-volume source of “bio-antibiotics,” with downstream purification achieved via selective chromatographic or affinity-based methods.

**Figure 6:**
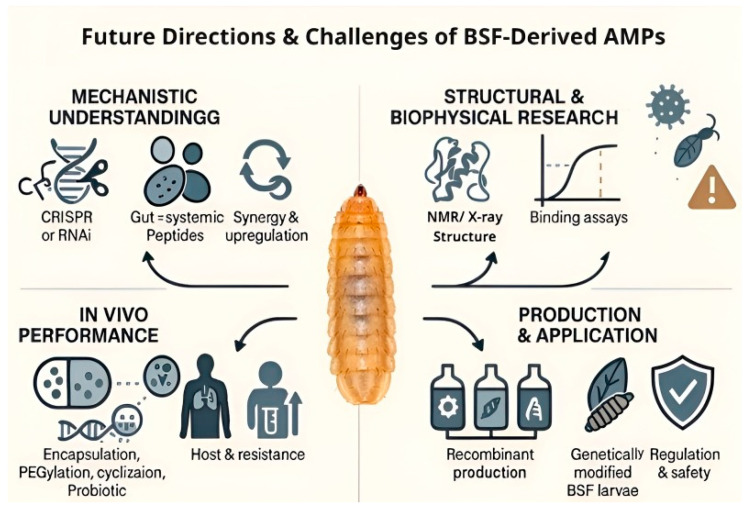
Mechanistic Understanding and challenges related to BSF-derived AMPs.created by a combination of software miro “https://miro.com (accessed on 1 Februray 2026)” and pixlr “https://pixlr.com (accessed on 1 Februray 2026)”.

Finally, regulatory and safety considerations remain pivotal. AMP deployment will require rigorous testing for immunogenicity, toxicity, and manufacturing consistency, alongside compliance with feed additive and pharmaceutical regulations. Figure 6 represents the key future aspects and challenges regarding BSF AMPs. Integrating these aspects will be essential for advancing BSF AMPs from laboratory discovery to real-world applications.

## 10. Conclusions

This review synthesizes current evidence on how BSF has evolved an unusually expanded AMP repertoire and how peptide physicochemistry, regulation, and mechanism jointly shape antimicrobial function in an AMR-relevant context. Overall, the literature supports the hypothesis that BSF AMP diversification is driven by pathogen-rich ecology and gene–family expansion, and that family-specific physicochemical constraints (disulfide-stabilized CSαβ folds in defensins; amphipathic helices in cecropins; β-architecture in attacins) are the main determinants of antimicrobial function. AI tools are most valuable when used to rank candidates and guide design, but only a standardized experimental pipeline can convert these principles into AMR-relevant leads. Across the literature, three consistent trends emerge. First, the BSF’s microbe-rich ecological niche plausibly drives recurrent gene duplication alongside balancing/diversifying selection, yielding large defensin- and cecropin-like paralog families with modest sequence changes that can shift activity spectra. Second, AMP deployment is highly plastic: constitutive “immune priming” and strong challenge-, tissue-, developmental-, and diet-dependent induction patterns indicate that regulation is a central determinant of when and where specific effector families operate. Third, the most defensible SARs are family-specific: defensin-like peptides are dominated by disulfide-stabilized fold constraints and cationic surface topology; cecropin-like peptides largely follow amphipathic α-helical design principles (net charge, amphipathicity/hydrophobic moment, hinge integrity) with clear selectivity trade-offs; attacin-like peptides represent a distinct β-architecture regime where classic small-AMP heuristics (e.g., “more charge”) are less predictive; and diptericin-/proline-rich peptides remain the most mechanistically under-resolved in BSF, with intracellular-targeting narratives largely inferred from other insects. In parallel, AI-enabled discovery (classifier-based prioritization and structure prediction) has accelerated candidate identification and hypothesis generation, but it cannot substitute for validation of processing/PTMs, stability in physiologically relevant matrices, selectivity, and mechanism; therefore, evidence-tiering (predicted → expression-supported → function-/mechanism-supported) is essential to prevent mechanistic overreach.

Major gaps that currently limit translation are methodological heterogeneity and incomplete BSF-specific mechanism mapping. Priorities that directly address the review objectives are: (i) standardized MIC/MBC and time–kill workflows with explicit assay conditions, molecular-weight–normalized units, peptide identity/purity/PTM reporting, and routine salt/serum/protease challenges; (ii) orthogonal mechanistic assays under physiologically relevant constraints to resolve whether killing is membrane-centered, pore-associated, or uptake-dependent for specific BSF peptides; (iii) causal genetics (RNAi/CRISPR perturbation of *Toll*/*Imd*/JAK–STAT nodes and AMP loci) to move from induction correlations to functional necessity and redundancy quantification; (iv) resistance-risk assessment as a required development variable (serial passage/experimental evolution, tolerance/biofilm assays, cross-resistance mapping) paired with mitigation-by-design strategies (combinations/cocktails and exposure minimization); and (v) translational engineering addressing stability, delivery/formulation, selectivity, manufacturability, and regulatory readiness. If these gaps are closed, BSF AMPs can progress from promising bioinformatic and *in vitro* leads to reproducible, mechanism-grounded candidates with credible potential for AMR-mitigation applications in therapeutics and antimicrobial-adjacent biotechnologies (e.g., functional feed and veterinary contexts).

## Figures and Tables

**Table 1:** SAR summary of representative BSF AMPs (DLP1–4, CLP1–3).

Peptide	Mature Sequence	µHα/µHβ (δ = 100°/160°, w = L)	Antimicrobial Activity MIC = MRSA/*E. coli*	Reference
Defensin-like peptide 1	ATCDLLSPFKVGHAACALHCIALGRRGGWCDGRAVCNCRR	0.215/0.181 (w = 40)	NR/NR	NCBI GenBank (accession KF805347.1)
Defensin-like peptide 2	ATCDLLSPFKVGHAACALHCIAMGRRGGWCDGRAVCNCRR	0.211/0.174 (w = 40)	0.12 µM/>29.97 µM	NCBI GenBank (accession KF805348.1) [43]
Defensin-like peptide 3	ATCDLLSPFGVGHAACAVHCIAMGRRGGWCDDRAVCNCRR	0.239/0.122 (w = 40)	5 µg per mL/10 µg per mL	NCBI GenBank (accession KF805349.1) [87]
Defensin-like peptide 4	ATCDLLSPFKVGHAACAAHCIARGKRGGWCDKRAVCNCRK	0.171/0.137 (w = 40)	0.59–1.17 µM/ND	NCBI GenBank (accession KF805350.1) [25]
Cecropin-like peptide 1	VFKPVEKFGQRVRDAGVQGIAIAQQGANVLATARGGPPQQG	0.331/0.124 (w = 41)	ND/0.52–1.03 μM	NCBI GenBank (accession JX997953.1) [52]
Cecropin-like peptide 2	VFKPVEKLGQRVRDAGIQGLEIAQQGANVLATARGGPPQQG	0.354/0.128 (w = 41)	NR/NR	NCBI GenBank (accession KF805345.1)
Cecropin-like peptide 3	VFKPVERLGQRVRDAGIQGLEIAQQGANVLATVRGGPPQQG	0.379/0.163 (w = 41)	NR/NR	NCBI GenBank (accession KF805346.1)

Note: μH values are not reported in the cited papers; they were calculated here to support SAR discussion and should not be interpreted as experimental measurements computed using EMBOSS hmoment with the window set equal to the mature peptide length (40 aa for DLP1–4; 41 aa for CLP1–3). Calculations were performed under α-helical periodicity (δ = 100°) and β-strand periodicity (δ = 160°), and the resulting whole-peptide µH values are reported as µHα and µHβ, respectively. L = Length, NR = not reported, ND = not detected.

**Table 4:** Examples of in silico predictions matched with experimental observations for BSF AMPs.

In Silico Prediction & Tool	Experimental Validation (Outcome)	Reference
Ampir prediction of Jg7904.t1 High AMP likelihood score (0.952) for a novel peptide in BSF genome. Model predicted a defensin-like sequence with 6 cysteines.	Identified *in vivo*: Jg7904 peptide detected in BSF larval hemolymph post-infection. Recombinant expression & assay: The peptide (approximately 7 kDa) exhibited antibacterial activity, notably inhibiting the growth of *P. aeruginosa*. Supports antimicrobial function under the tested conditions, active against Gram-negatives.	[12]
AlphaFold structural model of BSF attacin (Hill_BB_C10074) Predicted homotrimer forming a β-barrel pore (electrostatic and hydrophobic patterns indicating membrane insertion).	Membrane disruption confirmed: Recombinant attacin caused rapid killing of *P. aeruginosa*. TEM imaging revealed cell envelope damage, and impedance spectroscopy indicated the formation of pores in the membranes, is consistent with a pore-associated model, but does not establish oligomeric architecture.	[12,54]
Predicted disulfide connectivity in BSF defensin DLP4 [I-TASSER & DISULFIND] Model predicted Cys pairings in DLP4 matching insect defensin motif and a cationic surface patch.	Chemical validation: Mass spectrometry of recombinant DLP4 confirmed the three disulfide bonds in the predicted pairing (via reduction/alkylation patterns). Functional assay: DLP4 showed expected anti-*S. aureus* activity, supporting that the correctly folded (disulfide-intact) peptide is required for function.	[93,114]
Machine-learning multi-property prediction (CAMPR3) identified 57 peptides from BSF predicted to have antimicrobial (and some also antifungal/anticancer) activity using SVM/ANN models.	Synthesis and testing: A subset of these peptides was chemically synthesized. Several showed broad antimicrobial activity *in vitro* (matching predictions), while a few showed no activity (false positives). One peptide predicted to be antifungal did inhibit *Candida* growth in assays, validating the model’s suggestion.	[34,115]
Machine-learning multi-property prediction (CAMPR3): The C-15867 peptide sequence, VTCDLLKPFFGRAPCMMHCILRFKKRTGFCSRQN-VCVCR, was identified through a combination of transcriptomics and bioinformatics approaches.	Synthesis and Testing: The recombinant GST peptide fusion product was expressed in *E. coli* BL21 and *S. aureus* cells.	[93]

Note: AlphaFold-predicted structures can be accurate for many folded proteins but may be less reliable for short, flexible, or membrane-active peptides and do not by themselves establish oligomerization state or membrane-bound conformations. Docking provides plausible poses and relative ranking, but scoring does not constitute evidence of binding affinity or *in vivo* mechanism. ML AMP predictors can be biased by training set composition and negative-data selection; high scores should be interpreted as prioritization signals rather than functional proof.

**Table 5:** BSF Predicted Antimicrobial Peptides using Artificial intelligence.

Predicted Peptide	Why Biologically Prioritized	Evidence Grade (L1–L5)	References
Jg7904.t1 (defensin-like)	Predicted AMP and detected after infection; recombinant activity reported	L3 → L4 (function-supported; partial mechanism inference)	[12]
Hill_BB_C10074 (attacin-like)	Prediction linked to direct mechanistic assays	L4 (mechanism-supported; structure still inferred)	[54]
DLP4 (defensin-like; disulfide mapping example)	Structural prediction anchored to biochemical validation + function	L4 (→ L5 partial)	[88]
C-15867	Identified via transcriptomics + computational screening; sequence features suggest defensin-like AMP	L1–L2 (prediction/expression without clear functional endpoint in your text)	[93]

## Data Availability

No new data were created or analyzed in this study. Data sharing is not applicable to this article.

## Abbreviations

AMP(s)	Antimicrobial Peptide(s)
AMR	Antimicrobial Resistance
APD3	Antimicrobial Peptide Database 3
APD-3	calculator (within APD3) tool to compute physicochemical indices (hydrophobicity, Boman index, etc.)
AVPpred	Antiviral Peptide Predicto
BSF	Black Soldier Fly (*Hermetia illucens*)
CAMPR3	Collection of Anti-Microbial Peptides Resource 3
CFU	Colony-Forming Unit(s)
CFU/g	Colony-Forming Units per Gram
CLP	Cecropin-Like Peptide
CSαβ	Cysteine-Stabilized αβ Fold
DLP	Defensin-Like Peptide
DNA	Deoxyribonucleic Acid
e.g.	*exempli gratia* (for example)
*E. coli*	*Escherichia coli*
FDR	False Discovery Rate
FPKM	Fragments Per Kilobase of transcript per Million mapped reads
F-Value	(in kinetics) fraction of peptide–lipid binding vs. total (implicit in SPR/ITC data)
F-β-Fold	shorthand for Cysteine-stabilized β-sheet fold (CSββ)
*IMD*	Immunodeficiency pathway
i.e.	id est (that is)
ITC	Isothermal Titration Calorimetry
kDa	Kilodalton
LPS	Lipopolysaccharide(s)
MD	Molecular Dynamics
MIC	Minimum Inhibitory Concentration
MRSA	Methicillin-Resistant *Staphylococcus aureus*
NF-κB	Nuclear Factor Kappa-B
Omp	Outer Membrane Protein A
PRP	Proline-Rich Peptide motif
RNA	Ribonucleic Acid
RNA-seq	RNA Sequencing
RNAi	RNA Interference
*S. aureus*	*Staphylococcus aureus*
*S. cerevisiae*	*Saccharomyces cerevisiae*
SbmA	Bacterial inner-membrane peptide transporter
SEM	Standard Error of the Mean (implicit in some data plots)
SPR	Surface Plasmon Resonance
TEM	Transmission Electron Microscopy
*Toll* pathway	Insect pattern-recognition pathway activated by fungal β-glucans/Gram-positive peptidoglycan
α-Helical	shorthand for α-helical secondary structure
β-Sheet	shorthand for β-sheet secondary structure
